# New Insights for BPIFB4 in Cardiovascular Therapy

**DOI:** 10.3390/ijms21197163

**Published:** 2020-09-28

**Authors:** Marta Dossena, Anna Ferrario, Valentina Lopardo, Elena Ciaglia, Annibale Alessandro Puca

**Affiliations:** 1 Cardiovascular Research Unit, IRCCS MultiMedica, 20138 Milan, Italy; anna.ferrario@multimedica.it; 2 Department of Medicine, Surgery and Dentistry “Scuola Medica Salernitana”, University of Salerno, Via Salvatore Allende, 84081 Baronissi, Italy; v.lopardo@studenti.unisa.it (V.L.); eciaglia@unisa.it (E.C.)

**Keywords:** cardiovascular disease, aging, BPIFB4

## Abstract

Aging is the most relevant risk factor for cardiovascular diseases which are the main cause of mortality in industrialized countries. In this context, there is a progressive loss of cardiovascular homeostasis that translates in illness and death. The study of long living individuals (LLIs), which show compression of morbidity toward the end of their life, is a valuable approach to find the key to delay aging and postpone associate cardiovascular events. A contribution to the age-related decline of cardiovascular system (CVS) comes from the immune system; indeed, it is dysfunctional during aging, a process described as immunosenescence and comprises the combination of several processes overpowering both innate and adaptative immune system. We have recently discovered a longevity-associated variant (LAV) in bactericidal/permeability-increasing fold-containing family B member 4 (BPIFB4), which is a secreted protein able to enhance endothelial function through endothelial nitric oxide synthase (eNOS) activation and capable to protect from hypertension, atherosclerosis, diabetic cardiopathy, frailty, and inflammaging. Here, we sum up the state of the art of the mechanisms involved in the main pathological processes related to CVD (atherosclerosis, aging, diabetic cardiopathy, and frailty) and shed light on the therapeutic effects of LAV-BPIFB4 in these contexts.

## 1. Introduction

Cardiovascular diseases (CVDs) are the leading cause of mortality and morbidity of the over 65 years old population. From 2030, about 20% of the population will be 65 years old or older and 40% of these will die due to CVD [1]. In this scenario, the discovery of new therapeutic approaches to treat CVD is a clinical unmet need. CVD risk factors include environmental ones (i.e., alcohol and tobacco use, sedentary lifestyle, unhealthy diet) and other diseases, such as diabetes and immunological disorders [2,3,4]. Progressive aging of the population associates with frailty state, thus representing the most relevant risk factor in CVDs. Aging is a consequence of the increase of the life expectancy. In this context, aged individuals are clearly more exposed to age-related events such as hypertension, diabetes, and high level of cholesterol and obesity. [5]. Thus, even if people could live longer, this does not necessarily mean a longer disease-free life [6].

Aging is a physiological process that can be delayed, but not erased. In particular, this process works on vessels in different ways. Indeed, aged vessels show an impaired structure and an increase of stiffness and frailty. On the other hand, progenitors and stem cells display a significant inefficiency that translates in an impaired vascular regeneration [7].

Moreover, aging leads to a broad transformation in the whole cardiovascular system [8]. For this reason, understanding the molecular mechanisms responsible for the age-related vascular system decay is mandatory, in order to prevent the onset of both acute (myocardial infarction) and chronic (atherosclerosis) diseases.

Bactericidal/permeability-increasing fold-containing family B member 4 (BPIFB4) is a secreted protein that has been identified on a genome wide association study, in an Italian set of long-living individuals (LLIs) and controls. This association was validated also in two independent populations from Germany and the USA, that showed consistent enrichment in LLIs under recessive model of the minor allele rs2070325 (I229V) of BPIFB4. The rs2070325 is part of a four SNPs haplotype that codifies for a Wild Type (WT), a longevity-associated variant (LAV) with a 29% allele frequency, and a rare Variant (RV) haplotype of BPIFB4, the latter being present in 5% of chromosomes [9]

Here, we summarize and discuss the state of the art of the mechanisms involved in atherosclerosis (ATS), aging, and frailty, as relevant orchestrators of CVD, and the effects of LAV-BPIFB4 in these contexts. To optimize the state of art, we searched in PubMed database for published studies over the period 2000–2020. The following keywords were used to generate a search: BPIFB4, aging, frailty, atherosclerosis, type 2 diabetes, and inflammaging.

## 2. Centenarians as a Model to Escape Aging

As a consequence of the increase in life expectancy recorded in the last 160 years, more and more individuals reach an age of 100 years and over, now approaching 1 in 5000 individuals. This trend is explained by an improvement in hygienic conditions, reduced exposure to pathogens, and an improvement in the quality of life, including diet regimen. In particular, in favor of the diet as a longevity modulator, the elderly prospective cohort study (EPIC) has observed a decrease in mortality in those elderly people who consume the Mediterranean diet with a replacement of saturated fatty acids with monounsaturated ones [10].

Centenarians are able to reach more easily older ages than other individuals in the population, despite being exposed to the same environmental stimuli. In addition, they also show a decrease in morbidity and mortality, as a consequence of the demographic pressure. From a genetic point of view, this compression is correlated with an increase in the protective alleles that run into families, as demonstrated by an observed familiar clustering of exceptional longevity [11]. Indeed, centenarians’ children are more likely to reach extreme ages than their peers and show a decreased incidence of age-related pathologies [12]. Therefore, the world population of centenarians represents a very interesting genomic model and the analysis of the mechanisms that lead to longevity can be a great tool for understanding healthy aging [13].

Over the years, genetic analysis approaches, in particular GWAS (genome wide association study), have made possible the identification of genetic variants associated with longevity that confer an advantage in terms of survival [13].

An example is the epsilon 4 allele of the ApoE gene which is associated with an increase in CVD and Alzheimer disease, and which—in fact—is reduced in centenarians. On the other hand, a protective role of ApoE epsilon 2 has been revealed in a recent meta-analysis [14]. The ApoE knock out develop atherosclerosis, and this could explain the genetic association with exceptional longevity [15].

In other family-based studies, a locus at chromosome 4q25 has been linked with extreme longevity [11]. In this locus reside two genes, ELOVL6 and Gsnor, involved in disease resistance and aging [16]. Indeed, ELOVL6 knock out confers protection from diabetes while Gsnor regulates mitochondrial activity [17]. Furthermore, ELOVL6 has a role in modulating unsaturated fatty acids such as palmitoleic acid, whose levels have been found increased in the erythrocyte membranes derived from centenarians’ offspring as compared to age-matched septagenarians’ offspring [18]. ApoE2, instead, has been associated with a lower risk of developing atherosclerosis [14,19,20].

In 2011, the authors performed a GWAS on centenarians and controls enrolled in southern Italy, identifying 67 SNP passing a threshold of *p* < 0.0001 and candidate to be involved in determining the long-lived trait. Among these top findings, only four SNPs where missense polymorphisms, and of these only rs2070325 was replicated in two independent populations reaching statistical significance.

This missense mutation is located within the gene that codifies for bactericidal/permeability-increasing fold-containing family B member 4 (BPIFB4) protein [9].

## 3. Characterization of Longevity Associated Variant of Bactericidal/Permeability-Increasing Fold-Containing Family B Member 4

Bactericidal/permeability-increasing fold-containing family B member 4 (BPIFB4) is expressed in olfactory epithelium, mononuclear cells, germinal line, stem cells, progenitors, and fetal cells. This protein is composed by a regulatory domain located in N-terminal portion that contains phosphorylation and binding sites and by two big lipid binding pockets [21].

To date, three isoforms of this gene are known: the first isoform, named LAV-BPIFB4 is constituted by the minor allele of rs2070325 and in linkage disequilibrium with the other three SNPs (rs2889732, rs11699009, and rs11696307) [9]. The other two haplotypes are the wild-type (WT)-BPIFB4, which is constituted by major alleles of the 4 SNPS, and the rare variant (RV)-BPIFB4, which is generated by an event of recombination. Indeed, this haplotype is present only in 4% of the chromosomes, and is generated by a chimera of the WT and LAV-BPIFB4 retaining only the polymorphisms in position 3 and 4 of the LAV isoform. This rare variant (RV-BPIFB4) has been characterized by Vecchione et al. [22] in a population-based study whereby the authors observed that RV haplotype is associated with high blood pressure. Accordingly, mice treated with adenoviral vector carrying RV-BPIFB4 developed hypertension [23].

**Figure 1:**
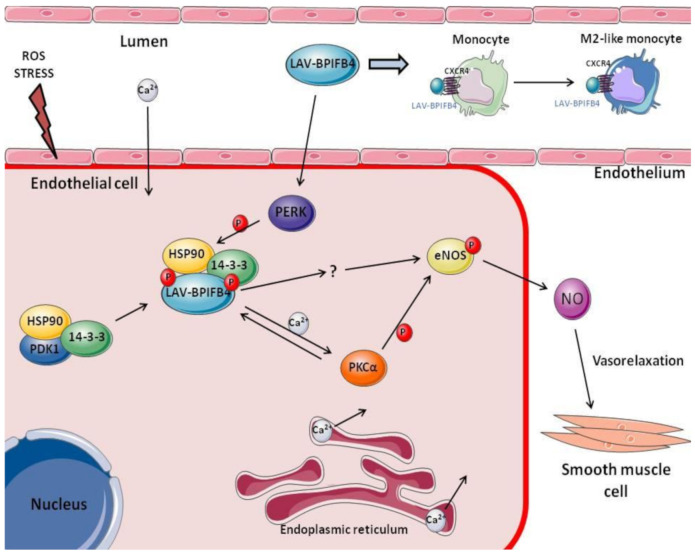
LAV-BPIFB4 mechanism of action. Schematic representation of LAV-BPIFB4 role in different pathways in endothelial cells and immune compartment. LAV-BPIFB4 is localized into EC cytoplasm that improves its phosphorylation by PERK and association with HSP90 and 14-3-3. This complex induces eNOS phosphorylation and NO production, leading to vasorelaxation in smooth muscle cells. On the other hand, LAV-BPIFB4 showed a polarizing effect on the phenotype of macrophages.

WT-BPIFB4 and LAV-BPIFB4 have different subcellular localization. In particular WT-BPIFB4 shows a nuclear localization, while LAV-BPIFB4 is cytoplasmic [9]. It makes LAV-BPIFB4 more prone to protein kinase R (PKR)-like endoplasmic reticulum kinase (PERK) dependent phosphorylation, a kinase involved in the unfolded protein response, pointing at LAV-BPIFB4 involvement in reducing endoplasmic reticulum stress and improving cellular homeostasis [9]. Furthermore, cytoplasmic localization of LAV-BPIFB4 is due to improved phosphorylation at Ser75 of BPIFB4, which allows interaction with 14-3-3 protein, which retains proteins in the cytoplasm, and Heat Shock Protein 90 (Hsp90). Ser 75 phosphorylation is due to PERK [9]. The BPIFB4-HSP90-14-3-3 complex is able to induce eNOS phosphorylation in Ser1177 resulting in NO production in endothelial cells. Moreover, LAV-BPIFB4 is phosphorylated by protein kinase C alpha (PKCα) in Ser75, which activates calcium mobilization, which in turn determines PKCα activation, generating a feed-forward mechanism. (Figure 1) [24].

## 4. BPIFB4 as a New Genetic Marker of Frailty

The term “frailty” was born in 2001, when Fried and colleagues published the work “Frailty in Older Adults: Evidence for a Phenotype”. Frailty is defined by geriatricians as a syndrome characterized by reduction in the reserve and resistance, both to stimuli exogenous and endogenous stress induction, due to the decline of more physiological systems which leads to an increased vulnerability to adverse outcomes. Despite a strict definition of frailty still represents a debated issue, Fried et al. in Cardiovascular Health Study first proposed some basic clinical manifestations characterizing frailty, then made operational as a validated instrument (i.e., frailty phenotype) [25]. Rockwood et al. in the Canadian Study of Health and Aging developed and validated the so-called Frailty Index [26]. This index describes the accumulation of deficits that occur with aging [27]. The main difference between frailty phenotype and frailty index is that this latter is based on the results of a comprehensive assessment of a patient and objectivizes a status of a biological aging [28]. Over the years, other models have been proposed and developed, but still based on the models by Fried and Rockwood. The definition of a frailty index is based on a set of five predominant criteria in which the presence/absence of signs or symptoms is assessed (i.e., involuntary weight loss, exhaustion, slow gait speed, poor handgrip strength, and sedentary behavior). The number of criteria (a six-level ordinal variable ranging from 0 to 5) is categorized into a three-level variable depicting robustness (none of the criteria), pre-frailty (one or two criteria), and frailty (three or more criteria). The frailty phenotype model does not require preliminary clinical evaluation and therefore can be used as a first approach to the patient [28] and could serve as an initial risk stratification of the population according to different profiles (i.e., robust, pre-frail and frail). In particular, the definition of “frailty status” has been demonstrated to be very useful in the field of cardiovascular disease to enhance the possibility to predict worse events after acute cardiovascular events [29,30]. Gerontological research, in recent years, has focused on recognizing and understanding the biological characteristics and pathophysiological determinants of age-related frailty. In particular, researchers are trying to identify biological markers that can be used for early screening (National Institute on Aging 2003). Research has recently focused on the molecular determinants of frailty such as chronic inflammatory states, that can cause an increase in interleukin 6, reduction of hemoglobin and hematocrit [31]; hormonal deficiencies (in particular IGF-I, DHEA-s) connected to a possible alteration immune system, despite the lack of a sure causal relationship [32,33]; changes in gene expression and shortened telomeres [34]; and reduction of the body’s ability to correct itself due to the loss of effectiveness of complex systems (reduction of complexity) [35].

Recently, Malavolta et al. showed that BPIFB4 haplotypes co-segregate with frailty and, subsequently, in order to obtain functional evidence of this association, gene therapy with LAV-BPIFB4 was used in a murine model of aging to counteract frailty. A cohort of 237 subjects belonging to the largest population described by Montesanto et al. with an age between 65 and 90 years was selected [36]. Patients were followed up by geriatricians who administered tests to assess frailty, and a frailty index that calculates the accumulation of deficits has been chosen. At the time of recruitment, subjects resulted 51% non-frail and 49% frail. Subsequently, genotyping for BPIFB4 haplotypes has been performed and the association analysis showed that carriers for LAV-BPIFB4 homozygous are underrepresented in the frails, as opposed to RV-BPIFB4 haplotype which is more frequent in the frail subjects [37].

Furthermore, following a cox regression analysis, a decreased survival rate was observed in carriers of the RV genotype.

To validate the association of the BPIFB4 genotype with the fragile phenotype at a functional level, a further study was conducted on adult and elderly mice in which LAV-BPIFB4 was administered as gene therapy. Interestingly, attenuation of clinical frailty has been reported following treatment with LAV-BPIFB4 in elderly mice [37]. This work, for the first time, has associated clinical evidence with experimental results whereby a gene haplotype may influence frailty. This outcome could lead to relevant advances in the clinic. On one side, the BPIFB4 haplotype could be employed as an early screening tool by geriatricians, to assess the risk of developing age-related frailty and disabilities, on the other side, LAV-BPIFB4 could represent a therapeutic molecule to counteract the symptoms in fragile patients.

## 5. LAV-BPIFB4: A New Approach for Atherosclerosis Treatment

The risk factors for ATS are well known and include hypertension, diabetes, high level of cholesterol, and smoke [38,39]. The mature atherosclerotic plaque is mainly composed by vascular smooth muscle cells (VSMC), collagen and elastin, that are produced by VSMC, inflammatory cells (macrophages, T lymphocytes, dendritic cells, NK cells, and mast cells) and by intra and extracellular lipid and debris [40]. Atherosclerotic plaque is surrounded by a fibrous cap that could exhibit diverse thickness and different inflammatory status; these two conditions regulate the plaque stability. The rupture of atherosclerotic plaque is due to VSMC death, the breaks of collagen and extracellular matrix and could lead to myocardial infarction or stroke [40]. In this context, LAV-BPIFB4 has been found to reduce ATS progression, as demonstrated in a population of 2606 people that was stratified for the presence of subclinical carotid ATS. In this population, it has been described that patients in absence of ATS display high serum level of BPIFB4; furthermore, genotype stratification analysis confirmed that LAV carriers significantly more frequently had an intima media thickness (IMT) < 2 mm and had a higher level of protective BPIFB4 compared with WT carriers [41].

LAV-BPIFB4 has been shown to exert a pleiotropic activity in different mechanisms involved in ATS. Its action affects different plaque compartments, but it is not associated with any lipid profile changes [41]. Despite LAV-BPIFB4 does not exert effect on the serum cholesterol profile, it is able to counteract the ability of oxidized cholesterol to induce endothelial dysfunction by positively modulating the ATS inflammatory environment [42].

During ATS, VSMC and endothelial cells (EC) enhance their secretion of pro-inflammatory cytokines/chemokines, such as IL-6, chemokines chemokine CC-motif ligand 2 (CCL-2), adhesion molecules (i.e., ICAM-1) and innate immune receptors (i.e., toll-receptor like 4; TLR-4) [43]. Also, VSMC proliferative rate is reduced in ATS [44]. In addition, increased number of senescent VSMC, EC, and monocyte/macrophage has been observed in atherosclerotic lesions [45,46]. Another key event occurring in ATS is apoptosis in fact smooth muscle and endothelial cells derived from atherosclerotic plaque show an increase of apoptosis [44,47]. All these features are also present in senescence, supporting the idea that ATS is associated with premature cellular senescence. Another important point that ATS and aging share is oxidative stress-induced damages in VSMC. This is a result of two critical aspects: a huge amount of reactive oxygen species (ROS) production and impaired antioxidant defense [48,49]. ROS production can modulate ATS progression both directly enhancing DNA damage in VSMC and EC [50,51] and influencing different mechanisms involved in premature cellular senescence. ROS are able to inhibit PI3K/Akt pathway that results in telomerase downregulation [52] and promotes apoptosis [53]. Additionally, ROS can enhance the expression of LDL uptake receptors, which has already described in aging process [54]. During ageing EC change their morphology becoming more flattened and enlarging with an increase of polypoid nucleus [55,56,57,58]. All these changes are accompanied by cytoskeleton modulations, proliferation, angiogenesis, and migration. Furthermore, senescent EC show reduced NO production [59] and release a high amount of endothelin-1 [60]. Moreover, it has been described a reduction of adhesion molecule VCAM-1 expression and intracellular adhesion molecules such as ICAM-1 in EC. On the other hand, pro-inflammatory molecules, such as NF-kB, are upregulated and cells are more liable to apoptosis [56]. EC senescence is associated to a progressive loss of their functionality and to a shift towards a proinflammatory and proapoptotic state [40].

In a mouse model of ATS (i.e., ApoE knockout mice fed a high fat diet), LAV-BPIFB4 is able to restore acetylcholine-mediated endothelial vasorelaxation in mesenteric and femoral arteries. Furthermore, increased expression and end phosphorylation status at Ser75 of BPIFB4 have been observed in the mesenteric artery [24]. In a similar way LAV-BPIFB4 is able to induce eNOS phosphorylation at Ser1177 and PKCα phosphorylation at Thr497 (Figure 1) [24]. In particular, eNOS is a key factor for CXCL12/CXC-chemokine receptor 4 (CXCR4)-mediated endothelial actions, as well as different PKC isoforms can mediate CXCR4 phosphorylation and activation [61].

Moreover, LAV-BPIFB4 is able to reduce plaques formation in ApoE knockout mice [41] and this effect is mediated by CXCR4. Moreover, it has been shown that CXCR4 and CXCL12 transcripts and protein expression are up-regulated in stable and unstable carotid atherosclerotic plaques, as compared to expression in young vessels [62]. However, it is not clear if the up-regulation of the CXCL12 /CXCR4 axis can act as a pathogenic or compensatory mechanism.

By ultra-structural analysis, LAV-BPIFB4 is able to preserve the physiological architecture of the vessel endothelium in a CXCR4-dependent manner [41]. Furthermore, LAV-BPIFB4 is able to reduce macrophages infiltration, without loss of VSMC and decreasing fibrous cap thickness [41]. It all points to a slowdown of the atherogenic process and to a plaque stabilization [63,64].

Altogether, these results pave the way to a new therapeutic approach for the treatment of ATS linking together LAV-BPIFB4 action with CXCL12/CXCR4 axis.

## 6. LAV-BPIFB4 as a Novel Treatment for Type 2 Diabetes Complications

Type 2 diabetes (T2D), first classified as ‘noninsulin dependent diabetes’ or ‘adult-onset diabetes’, represents 90–95% of all adult diabetes patients. T2D includes patients with absolute or relative insulin deficit and peripheral insulin resistance [65].

The rate of CVD in adult T2D patients is 2- or 3-fold higher than adults without diabetes and this is the leading cause of death for these patients [66]. Furthermore, the cost for diabetes treatment are ascribed to macrovascular and microvascular complications that cause myocardial stroke, hypertension, coronary artery disease, and peripheral vascular disease [67].

T2D is characterized by hyperglycemia, systemic insulin resistance and impaired cardiac insulin metabolic signaling. All these aspects are involved in diabetic cardiomyopathy [68]. In particular, diabetic cardiomyopathy is defined by the presence of impaired myocardial structure and function, without other cardiac risk factors, such as coronary artery disease, hypertension, and significant valvular disease in T2D patients [69]. In 2013, diabetic cardiomyopathy was defined as a clinical condition with ventricular dysfunction that occurs in absence of coronary ATS and hypertension in T2D patients. At the early stage of this pathology there is a hidden subclinical period characterized by structural and functional deficiencies that includes left ventricular (LV) hypertrophy, fibrosis, and impaired cell signaling. These pathophysiological changes of cardiac fibrosis and stiffness, combined with diastolic dysfunction, evolved to heart failure with normal ejection fraction and potential systolic dysfunction accompanied by heart failure with reduced ejection fraction [69].

In this context, the genetics of healthy longevity can pave the way to develop innovative treatment, in fact LLIs are protected from the consequences of age-related pathologies. BPIFB4 could be considered as an innovative drug to treat diabetic cardiomyopathy, because this protein is expressed in human heart and in particular in cardiomyocytes and endothelial cells. In heart failure, there is a down-regulation of BPIFB4 expression in cardiomyocytes, while in EC of coronary arteries there is no difference [70]. The efficacy of LAV-BPIFB4 treatment was evaluated also in diabetic mouse model (db/db mice), where BPIFB4 overexpression is able to protect the heart against diabetes-induced damage. LAV-BPIFB4 action shows a better positive effect than WT-BPIFB4. In particular, there is an increase of BPIFB4 expression in cardiomyocytes which is due to the uptake of circulating protein. Moreover, LAV-BPIFB4 is able to enhance diastolic function, preventing the increase in mitral valve deceleration time. In a similar way, LAV-BPIFB4 inhibits the increase of end-systolic volume, which is observed in db/db of 13–18 weeks old and improves systolic function [70]. At a cellular level, LAV-BPIFB4 is able to induce cardiomyocyte proliferation without changing their dimension or apoptotic rate. Furthermore, LAV-BPIFB4 improves MyHC-α expression and MyHC-α/ MyHC-β ratio in cardiomyocyte [70], counteracting the drop observed in diabetic heart [71]. The increase of MyCH isoform expression is strictly related to contractile property of individual myocytes [72] and restoration of endothelial function leading to an improvement of systolic performance after LAV-BPIFB4 treatment [70].

LAV-BPIFB4 efficacy is also observed in vessels, where it induces higher capillary density together with a reduction of lipid content and interstitial and perivascular fibrosis due to an increase of collagen degradation. This is able to prevent capillary rarefaction, fibrosis, and lipid accumulation that are normally seen in diabetic heart [70]. This effect together with the increase of eNOS phosphorilation lead to an improvement of vascular function as already demonstrated in hypertensive rats [9]. In this context, LAV-BPIFB4 is able to transiently reduce blood pressure [70].

LAV-BPIFB4 mechanism of action involves the activation of SDF-1/CXCR4 pathway. In fact, SDF-1 expression is enhanced in heart and PBMC of db/db mice and treatment with two CXCR4 antagonist counteracts LAV-BPIFB4 effects [70].

On the other hand, T2D significantly improves the risk of peripheral artery disease onset, which could lead to lower limb amputations. This pathology causes artery narrowing in limb resulting in tissue ischemia [73]. In particular, glucose level deregulation matched with oxidative stress enhancement, reduced NO production from eNOS that cause a less and slower revascularization process [74]. In this context, LAV-BPIFB4 is able to induce a significant recovery of superficial blood flow in a mouse model of limb ischemia improving capillary and arteriole density. Furthermore, LAV-BPIFB4 enhances VEGF and SOD3 expression in ischemic muscles suggesting LAV-BPIFB4 proangiogenic and scavenging actions [9].

## 7. The Role of LAV-BPIFB4 as an Immunoregulatory Driver in Age-Related CVD

Cellular senescence and inflammation are considered the major mechanisms governing aging process and age-related diseases, (ARDs) [75]. In this scenario, a persistent insult and time-dependent accumulation of cellular damags; the chronic release of senescence-associated secretory phenotype (SASP); and factors such as IL-6, RANTES/CCL5, IL-8/CXCL8, CXCL1, together with an impaired clearance and accumulation of senescent cells, lead to tissue dysfunction and chronic inflammation [76,77,78,79,80,81,82]. Cellular senescence involves adaptative immune system which shows an age-related functional impairment, termed immunosenescence [83]. Relevant features of immunosenescence include diverse processes overpowering both innate and adaptative immune system, such as SASP factors production by monocytes, decreased migration capabilities and reduced phagocytosis, thymic involution, decreased variability of the T cell receptor repertoire, lower number of B-cell precursors and plasma cell accumulation of expanded clones of memory and effector T cells [2,84,85]; furthermore, damaged mitochondria, extracellular ATP, uric acid, free cholesterol crystals, deposition of amyloid fibrils, ceramides, increased ROS levels, and accumulation of altered N-glycans with age were all observed. In this scenario, inhibition of autophagy and cell necropoptosis byproducts can chronically boost tissue resident innate immune cells (mainly macrophages) to trigger the low-grade chronic inflammatory response through inflammasome [86,87,88,89,90,91,92]. In this context, inflammaging can be considered the result of the imbalance between inflammatory and anti-inflammatory networks [93].

At the interface between innate and adaptive immunity, monocytes represent the keystone in instructing physiological homeostasis of the pro- and anti-inflammatory networks. Also, in the aging context, monocytes, the most abundant innate cells of myeloid origin, represent the underpinned yoke of a twin-pan balance where a compromised immune system and a chronic low-grade inflammatory status constitute the opposite weights. Monocyte phenotypes and functions are well established. In mice, two major monocyte subsets have been characterized, based on the different expression levels of the LyC6 surface antigen (i.e., classical LyC6^high^ monocytes and non-classical LyC6^low^ monocytes; [94]). In humans, the differential surface antigen expression of the CD14 and CD16 markers allows to discriminate three cell subsets (i.e., classical CD14^++^CD16^-^, non-classical ‘patrolling’ CD14^+^CD16^++^ monocytes and intermediate CD14^++^CD16^+^ monocytes that seem to have a mouse counterpart in Ly6C^++^CD43^++^ intermediate monocytes; [95]). Apart from migrating into tissues and lymphoid organs in steady-state condition and maturing into macrophages or dendritic cells, during inflammation, monocytes acquire additional functions both in humans and mice. They can patrol the vasculature, acting as antigen-presenting cells (APC) to T cells, thus priming adaptative immunity response, by supporting the instruction of T helper and promote Treg development [96]. Monocyte-derived macrophages can polarize into classically activated pro-inflammatory M1-like macrophages or alternatively activated anti-inflammatory M2-like macrophages, depending on stimuli and microenvironment [97]. Changes in monocytes–macrophages and monocytes–dendritic cells during aging are largely investigated [98] but some aspects of the driving force of these changes are still unknown.

The involvement of the immune system in vascular homeostasis and pathogenesis of CVD is well-known. Mononuclear phagocytic cells are critical components that act by patrolling vascular endothelium, oscillating between both a pro-inflammatory M1-like or pro-resolving M2-like state [99,100]. In this regard, the immune system remodeling/adaptation and the recovery of the inflammatory balance, occurring in LLIs, very likely regulated by LAV-BPIFB4, have led to intriguing pre-clinical evidence supporting an alternative therapeutic option in CVD management. In the previously described ATS mouse model [41], in response to the LAV-BPIFB4 infection it was observed a redistribution of circulating monocyte subsets, characterized by a reduction of Ly6C^low^ monocytes and an increase of Ly6C^high^ monocytes compared to control mice. As suggested by previous findings, LAV-BPIFB4 might perform its immunomodulatory activity in a CXCR4-dependent manner. Accordingly, it has been reported an increased percentage of CXCR4^+^Ly6C^high^ cells in the bone marrow, spleen and, partially, peripheral blood of ApoE^−/−^ mice, following LAV-BPIFB4 gene transfer, and confirmed by using a CXCR4 inhibitor (i.e., AMD3100) [101]. In this regard, LAV-BPIFB4 could trigger the monocytes tissue reservoirs orchestrating the monocytes mobilization into peripheral tissues, upon injury. Interestingly, LAV-BPIFB4 showed a polarizing effect on the phenotype of splenic macrophages that acquire a pro-resolving M2-like polarization state. As expected, this associated with a reduction of T-cell activation and proliferation via a CXCR4-dependent mechanism [41]. During ATS, EC undergo inflammatory activation that leads to the recruitment of leukocytes through adhesion molecules (e.g., VCAM-1) and their migration into intima through chemoattractant-receptor interaction (e.g., MCP-1/CCL2 and CCR2) [102]. Among leukocytes, the recruitment of Ly6C^high^ monocytes was found crucial in plaque regression, due to their trend to differentiate into M2-like macrophages [103]. Since mono-macrophages seem to represent a driving-force in this context, the LAV-BPIFB4-driven M2-like polarization can have a desirable protective potential in the resolution of the atherogenic process. The achieved results also have a translational significance considering that we also tested the effect of human recombinant LAV-BPIFB4 on CD14^+^ PBMCs from ATS patients that confirmed the CXCR4-dependent LAV-BPIFB4 pro-resolving polarizing effects, accompanied by the improvement of the inflammatory balance (e.g., reduced IL-1β and TNF-α levels and increased IL-33 levels) that can contrast the low-grade chronic inflammation affecting the vascular atherosclerotic process (Figure 1) [41].

From a mechanistic point of view, it has been recently found that the SDF-1/CXCR4 axis is also involved in the immunomodulatory action, mediated by LAV-BPIFB4 in diabetic cardiomyopathy, both in mice and humans. Accordingly, LAV-BPIFB4, but not WT-BPIFB4, is able to induce the expression of circulating SDF-1 in peripheral blood and heart of diabetic mice [70]. In addition, considering circulating mononuclear cells from T2D patients, in vitro LAV-BPIFB4 treatment leads to an up-regulated expression of SDF-1 uniquely in CD14^+^CD16^+^ intermediate monocytes (characterized both by inflammatory potential and as independent predictors of cardiovascular events by Zawada et al.) [70,100]. Even though the mechanism involved in SDF-1-induction mediated by LAV-BPIFB4 is still unknown, the relevance of SDF-1 in myeloid cell differentiation [104], tissue repair [105] and stem cell mobilization [106] could explain its protective immunoregulatory action against most of age-related and CVD.

Exceptional longevity associated not only with lower incidence of cardiovascular events [107] but also with preservation of some brain function [108] to properly escape neurodegenerative disorders such as Alzheimer’s disease and Parkinson’s disease [109]. As in cardiovascular system, also in aged brain a sustained inflammatory circuit often compromises cell viability and correlates with the degree of neurodegeneration. In this context, we have recently documented the ability of the LAV-BPIFB4 to activate key processes in striatum cell homeostasis both in in vitro and in vivo models of Huntington’s disease. In addition to ensuring the proper control of the cell cycle and viability of mutant striatal cells in response to the proteasome blocking insult, LAV-BPIFB4 overexpressing cells are able to educate co-cultured microglia to acquire an M2 (CD163^+^IL-10^high^) anti-inflammatory phenotype in vitro. From a mechanistic point of view, by inducing SDF-1 secretion by transduced cells, LAV-BPIFB4 gene transfer can blunt the unbalanced immune responses not only in peripheral tissues, but also in central nervous system [110] through a beneficial SDF-1/CXCR4 axis.

## 8. Conclusions

Aging accounts as of the most risk factor to the onset and progression of CVD, the leading cause of death in the western nations. Currently, aging induces cellular and molecular alterations affecting the endothelium, smooth muscle components, and immune system functions that drive the onset of CVD. In this context, LLIs represents a genomic model through which study the mechanisms that lead to longevity understanding healthy aging. Among these, LAV-BPIFB4 is able to finely tune endothelial function and the pro- and anti-inflammatory balance making this protein a promising candidate new ‘drug’ to treat ATS, its cardiovascular complications, and neurodegenerative diseases.

## 9. Patents

A.A.P. hold shares of LGV1 start-up, a spin-off of University of Salerno that own patents on LAV-BPIFB4 use in therapy.

## Abbreviations

APC	Antigen-presenting cells
ARDs	Age-related diseases
ATS	Atherosclerosis
BPIFB4	Bactericidal/permeability-increasing fold-containing family B member 4
CCL-2	Chemokines chemokine CC-motif ligand 2
CVDs	Cardiovascular diseases
CXCL-xx	Chemokine (C-X-C motif) ligand xx
EC	Endothelial cells
EPIC	Elderly prospective cohort study
GWAS	Genome wide association study
Hsp90	Heat Shock Protein 90
ICAM-1	Intercellular adhesion molecule-1
ILx	Interleukin x
LAV	Longevity-associated variant
LLIs	Long-living individuals
LV	Left ventricular
MCP1	Monocyte chemoattractant protein 1
PERK	Protein kinase R (PKR)-like endoplasmic reticulum kinase
PKCα	Protein kinase C alpha
RANTES	Regulated upon activation, normal T cell expressed and secreted
ROS	Reactive oxygen species
RV	Rare variant
SICS	Southern Italian centenarian study
SOD3	Superoxide dismutase 3
T2D	Type 2 diabetes
TLR-4	Toll-receptor like 4
TNFα	Tumor necrosis factor α
VCAM1	Vascular cell adhesion molecule 1
VEGF	Vascular endothelial growth factor
VSMC	Vascular smooth muscle cells
WT	Wild type
